# Internal Audit of an Oral Pathology Laboratory: Perspectives on Finances and Operational Management

**DOI:** 10.7759/cureus.70997

**Published:** 2024-10-07

**Authors:** Priyadharshini G, Karthikeyan Ramalingam, Pratibha Ramani, Deepak Nallaswamy

**Affiliations:** 1 Oral Pathology and Microbiology, Saveetha Dental College and Hospitals, Saveetha Institute of Medical and Technical Sciences, Saveetha University, Chennai, IND; 2 Prosthodontics, Saveetha Dental College and Hospitals, Saveetha Institute of Medical and Technical Sciences, Saveetha University, Chennai, IND

**Keywords:** biopsy, financial audit, hospital administration, income, lab administration, oral pathology, pathology lab administration, resource allocation, squamous cell carcinoma, total quality management

## Abstract

Background

Internal audits are essential tools for enhancing the operational efficiency, quality, and effectiveness of healthcare departments. Audits enable the departments and laboratories to meet the changing needs of the healthcare environment by giving a detailed picture of the department's operations and highlighting areas for possible growth and development.

Aims and objectives

This study focuses on the biopsies received in the Oral Pathology Department at Saveetha Dental College, aiming to evaluate biopsy trends, financial performance, and resource utilization over one year.

Materials and methods

The oral pathology department audit covered the period from 1st April 2023 to 31st March 2024. The institutional human ethical committee and scientific review board approved the retrospective audit. It involved a comprehensive analysis of biopsy data, financial records, and material usage. Data on different biopsy types (excisional, incisional, frozen sections), immunohistochemistry, cytology, and special stains were collected and analyzed across four quarters. Financial performance was assessed by comparing total income and expenses, while resource utilization was examined through the use of histopathological blocks and other consumables. Statistical analysis (chi-square) was performed using IBM SPSS Statistics for Windows, Version 23 (Released 2015; IBM Corp., Armonk, New York, United States). A P-value less than 0.05 was considered statistically significant.

Results

We received 1100 cases during the study period. Excisional biopsies were the most common, with 474 (43.09%) cases, followed by incisional biopsies with a total of 432 (39.27%). Out of total cases of 1100, the second quarter (July-September 2023) had the highest case volume of 305 (27.72%), while the third quarter (October-December 2023) recorded the lowest of 250 (22.72%) cases. A financial audit revealed an annual deficit of ₹1,03,321 primarily due to higher expenses towards laboratory reagents. The overall expense incurred per case was ₹448.5. Tissue blocks cost ₹85.23 per case (19.00%) of the average cost per case. The chi-square test analysis was insignificant among the different types of biopsies and the reagent consumption across the four quarters.

Conclusion

The audit identified critical areas for improvement in both clinical workload and financial management. High volumes of biopsies, but net financial deficits highlight the need for better cost management and resource utilization strategies to maintain sustainability without compromising diagnostic quality.

## Introduction

Internal audits are crucial for guaranteeing the efficacy, caliber, and efficient operation of specialty healthcare departments like oral pathology [1]. Health authorities and organizations prioritize audits as a means of identifying areas for improvement, implementing changes for the better, and rigorously evaluating the care that is delivered. This method is known as quality improvement [2]. These audits are an essential tool for locating inefficiencies and difficulties in operations, which helps departments keep up their high levels of care [3]. Extensive attention to detail is necessary in oral pathology for accurate diagnosis and treatment of diseases, including potentially malignant disorders and oral squamous cell carcinoma (OSCC) [4]. OSCC presents complex diagnostic challenges that require rigorous evaluation and continuous monitoring [5].

Internal auditing is defined as an impartial and independent activity that offers assurance and advisory services to an organization according to the International Professional Practices Framework (IPPF), 2017. Internal audits offer significant insights that support well-informed decision-making and strategic planning by methodically examining a variety of departmental performance metrics, including resource utilization, financial management, and case management [6]. An internal audit also evaluates the use of resources, including the number of tissue blocks and associated costs, ensuring that diagnostic processes are both cost-effective and of high quality. Furthermore, the audit helps identify trends in patient cases and financial performance, supporting better resource allocation and strategic planning. Departments can make sure they are maximizing their resources and providing high-quality diagnostic services by conducting internal audits regularly [7]. Through longitudinal analysis and a grasp of case management dynamics, departments can adjust to variations in caseload, optimize resource distribution, and ultimately improve patient outcomes [8,9]. Audits enable departments to stay flexible and responsive to the changing needs of the healthcare environment by giving a detailed picture of the department's operations and highlighting areas for possible growth and development [10]. An internal department audit can ensure the fulfillment of standards of care by methodically evaluating laboratory procedures and reagents used [11]. It comprises the application of effective audit, collection, and data analysis. Dissemination of such audit results can bring about effective change within the organization [10].

There is a lack of studies that concentrate on the operational aspects of oral pathology laboratories [12,13]. Studies require a longitudinal methodology to identify changes and patterns that take place over a longer period. In their study, Surendran et al. and G. P. et al. compared expenses over two years and evaluated turnaround time (TAT) for oral squamous cell carcinoma (OSCC) cases, respectively, which laid the groundwork for this research [14,15].

This manuscript focuses only on auditing the highly used materials within the oral pathology department, identifying their usage, attempting to optimize the material usage, and enhancing the quality of reporting. This study analyzed the biopsy data, purchase records, and resource utilization to comprehend oral pathology laboratory operations, especially OSCC samples.

## Materials and methods

An internal audit was conducted in the Oral Pathology Department at Saveetha Dental College, Chennai, India, from 1st April 2023 to 31st March 2024. This retrospective audit was approved by the institutional human ethical committee and scientific review board by letter number IHEC/SDC/PhD/O PATH-2212/22/001. The audit involved a comprehensive analysis of biopsy data, financial records, and resource utilization over the specified period for samples sent from procedures performed under local and general anesthesia. 

Data collection

Monthly biopsy data were collected and analyzed to identify trends in case volumes and types of biopsies performed. The audit focused on four quarters of the year, namely the First Quarter: April, May, and June 2023; the Second Quarter: July, August, and September 2023; the Third Quarter: October, November, and December 2023; and the Fourth Quarter: January, February, and March 2024.

The types of samples included were excisional biopsies, incisional biopsies, frozen section biopsies, immunohistochemistry (IHC), fine needle aspiration cytology, and special strains for procedures that were performed under local anesthesia and general anesthesia. Further, the total number of college cases and those cases referred from other private institutions were calculated.

A financial audit was performed to evaluate the economic performance of the pathology laboratory. The analysis assessed the total income from the cases processed and the total expenses incurred for materials. Income and expense calculation per case and overall financial deficit were analyzed.

The audit assessed the total number of tissue blocks prepared, the average number of blocks per case, and the cost per block. Further, the overall quantity of material usage along with the utilization of these materials per case was calculated. Laboratory reagents, including formaldehyde, ethanol, propanol, xylene, and acetone, were calculated. Other laboratory consumables including tissue cassettes, microscope slide boxes, microtome blades, Bard-Parker blades, paper sheets, and slide stickers were analyzed.

Exfoliative cytology samples and other samples were excluded from this internal audit. The salary expenditure of reporting pathologists, laboratory staff, and supporting maintenance staff was beyond the scope of this internal audit.

Statistical analysis

Data were aggregated, tabulated, and exported to Microsoft Excel (Microsoft Corporation, Redmond, Washington, United States). Statistical analysis was done in IBM SPSS Statistics for Windows, Version 23 (Released 2015; IBM Corp., Armonk, New York, United States). A descriptive analysis was performed. Chi-square tests were performed to analyze the difference in data between each quarter. A P-value less than 0.05 was considered statistically significant.

## Results

**Figure 1:**
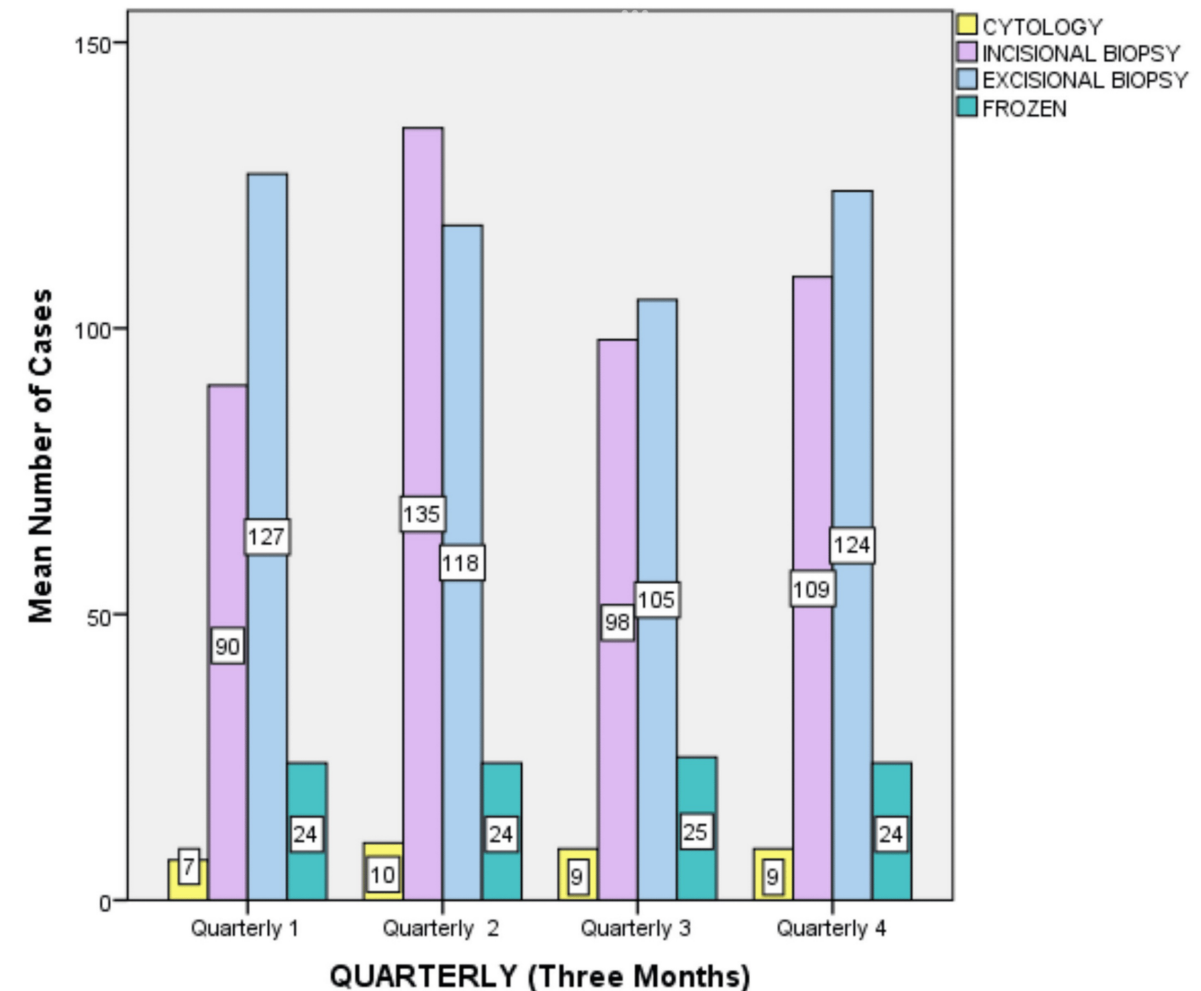
Graph showing variations in the number of each sample type received across four quarters.

The analysis of biopsy data from April 2023 to March 2024 revealed 1100 cases that were performed under local anesthesia and general anesthesia. Excisional biopsies were predominant, totaling 474 cases (43.9%), and it was highest in the first quarter (April, May, and June 2023). Incisional biopsies had 432 cases (39.27%) and frozen had 97 cases (8.81%), while cytology had 35 cases (3.18%) (Figure 1).

**Figure 2:**
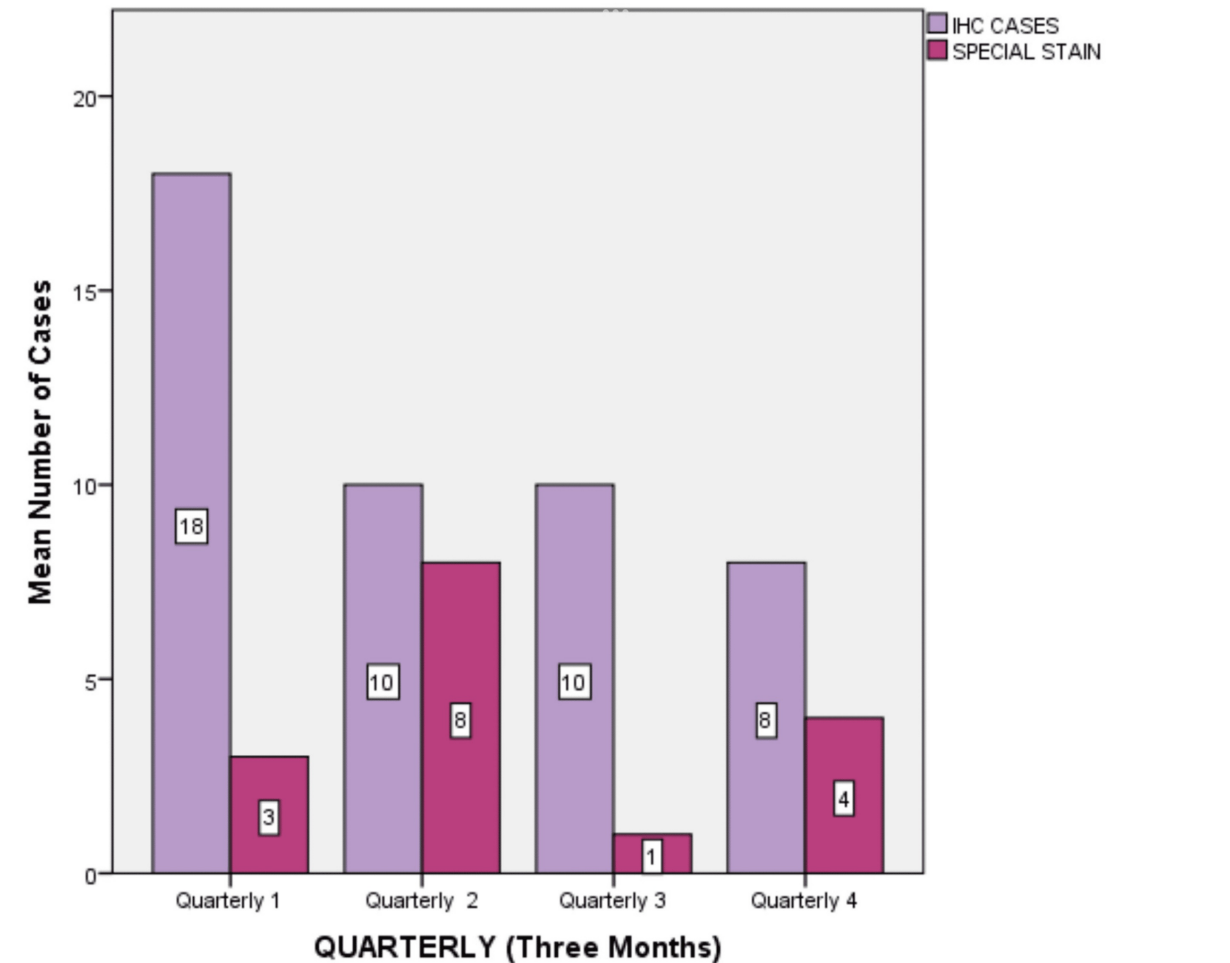
Graph illustrates the variations in immunohistochemistry (IHC) and special stain cases across different quarters.

There were differences in the number of cases across the four quarters. The second quarter (July, August, and September 2023) exhibited the highest case volume (n=305) which represented 27.72% of the total cases. In the second quarter, the cases in August showed 110 cases (10%). The third quarter (October, November, and December 2023) had 250 cases (22.7%) and was the lowest in the study. November month showed 62 cases (5%). Immunohistochemistry had 46 cases (4.18%), followed by special stains with 16 cases (1.45%) during the study period (Figure 2).

The chi-square test did not show statistical significance (p>0.005) in the number of cases (excisional, incisional, frozen, cytology, IHC, and special stains) among the four quarters. The p-value for cytology was 0.651, incisional biopsy was 0.330, and excisional biopsy was 0.368. Frozen cases showed a p-value of 0.333. Immunohistochemistry (IHC) and special stains had p-values of 0.333 and 0.534, respectively (Table 1).

**Table 1:** Chi-square test analysis of different cases among the four quarters. IHC: immunohistochemistry

Type of cases	P-value (Chi-square analysis)
Cytology	0.651
Incisional biopsy	0.330
Excisional biopsy	0.368
Frozen	0.333
IHC	0.333
Special stain	0.534

Out of total biopsy cases of 1100, OSCC cases were 244 cases, representing 22.18% of the total cases. Among these 244 OSCC cases, 166 (68.03%) were incisional biopsies and 78 (31.96%) were excisional biopsy cases. It was found that the total number of college cases (n=843, 76.63%) was higher compared to that of private referral cases (n=257, 23.36%). The financial audit revealed that for the 1,100 cases processed, the total income was ₹3,90,030, while the expenses were ₹4,93,351. A net loss of ₹1,03,321 (26.49%) was observed. The calculated overall income per case was ₹354.57, while the expenses per case were ₹448.50. The income in OSCC cases was ₹86,376 and that of their expenses was ₹1,09,312. In the current study, a total of 5,788 tissue blocks were found to be utilized across 1,100 cases, averaging 5.26 blocks per case. The cost associated with each tissue block was determined to be ₹85.23, which accounted for 19.00% of the overall average expense (₹448.5) per case.

Among the reagents analyzed from April 2023 to March 2024, propanol had the highest usage, with an overall average of 226 units and an average of 102.95 ml per case during the study period. Ethanol had the lowest total usage, with an overall average of 31 units and 14.06 ml used per case (Table 2).

**Table 2:** Laboratory reagents utilized for processing the received cases.

Month	Formaldehyde	Usage Per Case	Ethanol	Usage Per Case	Propanol (500ml)	Usage Per Case	Xylene (500ml)	Usage Per Case	Acetone (500ml)	Usage Per Case
April-2023	4	22.72	1	8.06	18	102.27	4	22.72	12	68.18
May-2023	3	17.64705882	5	29.41176471	16	94.11	5	29.41	9	52.94
June-2023	4	20.83333333	4	20.83333333	24	125	8	41.66	15	78.12
July-2023	5	25.77319588	3	15.46391753	17	87.62	4	20.61	8	41.23
August-2023	1	4.545454545	3	13.63636364	19	86.36	6	27.27	9	40.9
September-2023	4	20.40816327	1	5.102040816	23	117.34	4	20.4	9	45.91
October-2023	6	30.6122449	3	15.30612245	21	107.14	8	40.81	14	71.42
November-2023	3	24.19354839	1	8.064516129	12	96.77	5	40.32	8	64.5
December-2023	3	17.04	4	22.72	20	113.63	4	22.72	9	51.13
January-2024	6	32.6	5	27.17	18	97.82	7	38.04	9	48.91
February-2024	2	10.63	1	5.31	16	85.1	6	31.91	9	47.87
March-2024	5	27.77	0	0	22	122.22	7	38.88	14	77.77
TOTAL	46	21.23	31	14.06	226	102.95	68	30.9	125	57.41

**Figure 3:**
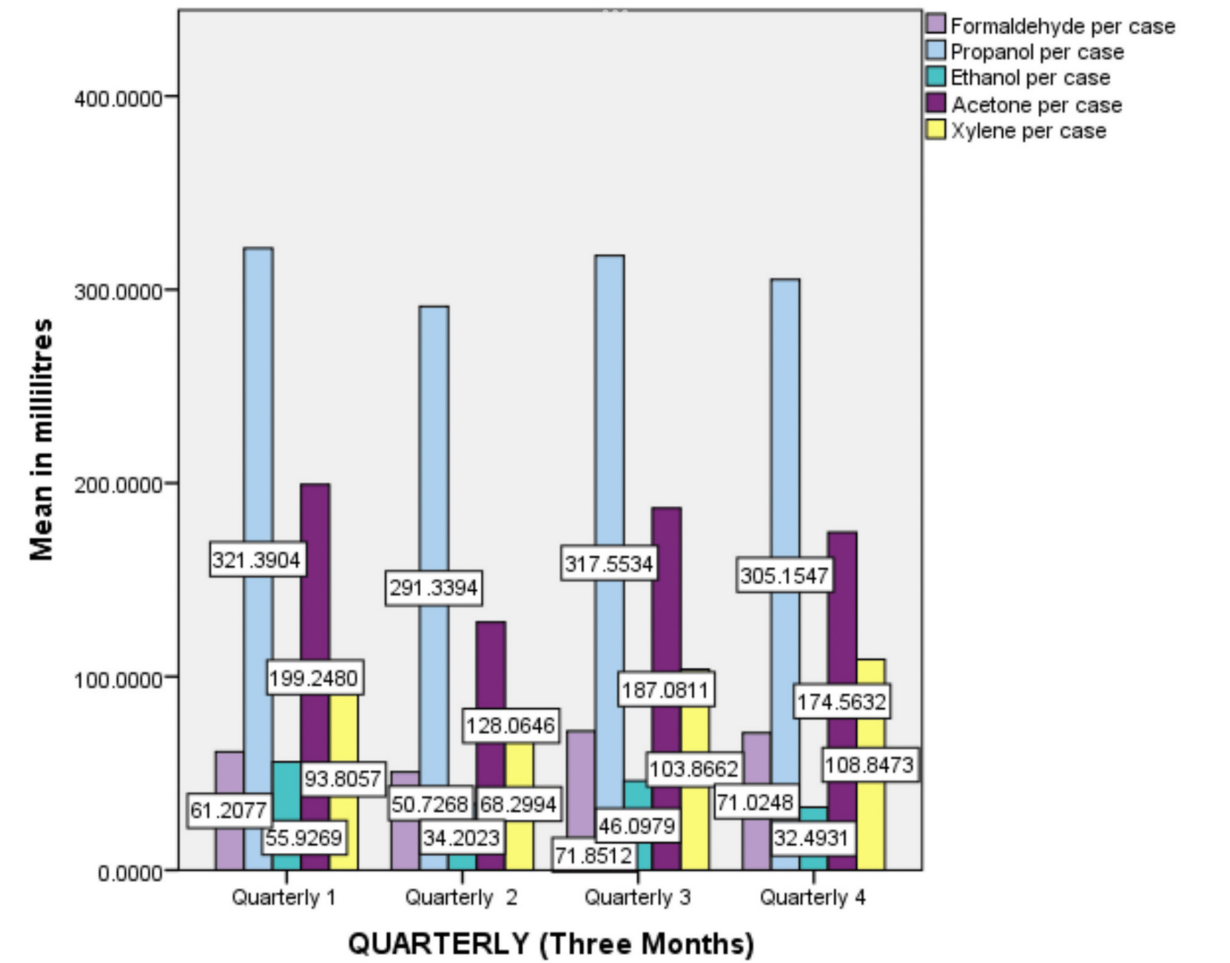
Graphical representation of the quarterly usage of laboratory reagents.

On average, 2.2 liters of formalin is the average for one excisional biopsy specimen, and 30 ml of formalin is the requirement for one incisional biopsy specimen. These results highlight that propanol was the most frequently utilized reagent. The highest usage of propanol was in the first quarter (321.39 ml), while ethanol was the least used reagent during the study period, which was high in the first quarter (55.92 ml), acetone was highly used in the first quarter (199.24), and high usage of xylene was in the fourth quarter (128.06) (Figure 3).

The total usage of laboratory supplies from April 2023 to March 2024 was evaluated. Tissue cassettes had the highest total usage of 6275 units, with an average of 5.78 units per case. Microslide boxes (50-count) showed a total usage of 199 boxes, with 10.08 slides per case, while slide stickers had a total usage of 143 sheets, with an average usage of 20.49 stickers per case (Table 3).

**Table 3:** Laboratory supplies consumption for the study period.

Month	Tissue Cassettes	Usage Per Case	Microslide Box (50)	Usage Per Case	Slide Stickers	Usage Per Case
April-2023	409	4.64	5	2.84	14	24.81
May-2023	490	5.764705882	15	8.823529412	10	18.35294118
June-2023	504	5.25	29	15.10416667	17	27.62
July-2023	455	4.690721649	14	7.216494845	12	19.29
August-2023	373	3.390909091	21	9.545454545	15	21.27
September-2023	634	6.469387755	19	9.693877551	13	20.69
October-2023	890	9.081632653	19	9.693877551	7	11.14
November-2023	477	7.693548387	21	16.93548387	11	27.27
December-2023	483	5.48	17	9.65	12	21.27
January-2024	615	6.68	17	9.23	13	22.04
February-2024	455	4.84	-	-	11	18.25
March-2024	490	5.44	22	12.22	8	13.86
TOTAL	6275	5.78	199	10.08	143	20.49

**Figure 4:**
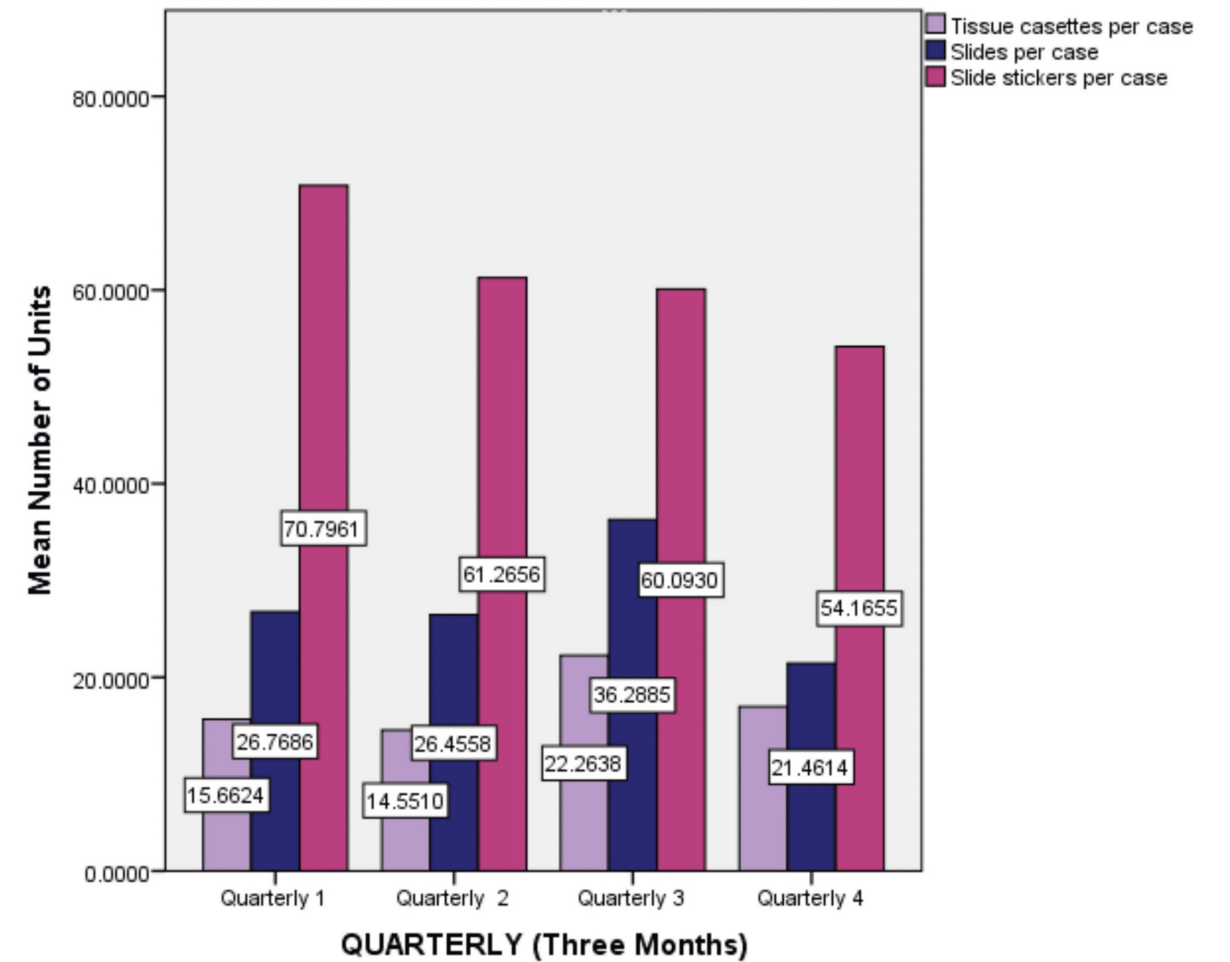
Graph showing quarterly usage of laboratory consumables.

The audit revealed an average of 70.79 slide stickers in the first quarter. Slide usage was highest in the third quarter with a total of 36.28 slides per case, and the tissue cassettes showed an average of 22.26 in the third quarter (Figure 4). 

It was found that none of the laboratory consumables showed statistically significant differences (p > 0.05) across the quarters. Among the reagents used per case, formaldehyde (p=0.081) and xylene (p=0.084) had the lowest p-values but were still above the significant threshold value. Propanol, ethanol, and acetone had p-values of 0.782, 0.330, and 0.290, respectively. Among the other consumables, tissue cassettes had a p-value of 0.299, slides had a p-value of 0.382, and slide stickers showed a p-value of 0.261. Chi-square analysis revealed p-values for the difference in reagents used among the four quarters given in Table 4.

**Table 4:** Chi-square analysis of different laboratory reagents and consumables across all quarters.

Reagents Used (Per Case)	P-value (Chi-Square Analysis)
Formaldehyde	0.081
Propanol	0.782
Ethanol	0.330
Acetone	0.290
Xylene	0.084
Other consumables (per case)	
Tissue cassettes	0.299
Slides	0.382
Slide stickers	0.261

A few items, like blades and paper, were distributed directly on request. Therefore, these were not included in our quarterly material consumption data. The annual data for these items during the study period include 300 Leica 818 microtome blades (Leica Biosystems, Deep Park, IL), 600 Bard-Parker blades, seven bottles of DPX mounting medium, 320 boxes of coverslips, 3287 sheets of A3-sized paper for grossing, and 3937 sheets of A4-sized paper for report printing.

## Discussion

This manuscript focuses only on auditing the highly used materials within the oral pathology department, identifying their usage, attempting to optimize the material usage, and enhancing the quality of reporting. Our study provides a comprehensive overview of the trends, patterns, and financial aspects of biopsy cases handled at our institution over a specific period. It was found that August was the peak month with the highest number of total cases, driven by a significant number of incisional biopsies and cytology cases. In November, the total number of cases declined. Jabbour et al. reported that oral cancer was more likely to be diagnosed in spring. The seasonal variation in biopsy volumes differs across different months, often influenced by academic schedules and climate changes across different countries [16]. Furthermore, medical professionals or clinicians might have specific referral patterns that increase during certain months. However, the lack of significant differences in the number and types of cases received in our institution reflects the constant inflow of cases over the entire year without any major variations.

OSCC comprised 22.18% of the total cases. This indicates the high number of OSCC case referrals to our institution. The initial diagnosis of OSCC cases was made with an incisional biopsy. Many of these cases were consequently operated on as excisional biopsies in our institution, attributing to the availability of intraoperative diagnosis and oral cancer management in our institution. Excisional biopsy for OSCC is essential as it offers crucial diagnostic data and establishes precise surgical margins for further treatment planning and follow-up protocols [17,18]. 

The majority of the cases were from our college (76%), while a smaller proportion were from private clinics (23%). Private clinics may have limitations in diagnostic facilities and the ability to handle complex cases, especially those involving potentially malignant or malignant lesions like OSCC [19]. As a result, they often refer such cases to larger institutions, where comprehensive diagnosis and treatment planning, including histopathological evaluation, immunohistochemistry procedures, and surgical management, can be provided. This referral pattern could have contributed to the higher number of cases received in our institution.

Furthermore, the financial analysis revealed that the overall expenditure was significantly higher than the income, with an overall deficit of ₹1,03,321 in one year. The increased operating expenditures in maintaining the oral pathology department in an academic university setting could be a major contributing factor [14,20]. The adoption of expensive procedures like immunohistochemistry and frozen sections could escalate costs. According to a study by Cheah et al., salaries make up the majority of the budget in pathology laboratories (40.3%), followed by consumables and reagents [21]. These findings highlight the need for effective financial management strategies to address financial deficits in laboratories [22]. Ensuring financial stability while maintaining the quality of education and patient care is a complex but achievable goal for academic health centers [23].

The wax blocks, making up 18% of the average expense per case, highlight the significant financial impact of wax block usage in histopathology. This reflects not only the direct costs associated with materials, such as paraffin wax and cassettes, but also the operational costs involving skilled technicians, equipment maintenance, and quality control measures [24]. Although the increased use of blocks might be necessary to provide thorough samples for diagnosis, it is important to balance their usage with cost-efficient practices to avoid financial deficits [25].

The high consumption of formaldehyde can be attributed to its essential role in the preservation and fixation of tissue specimens, which is critical for accurate diagnosis in oral pathology. The high usage of formaldehyde emphasizes its importance in maintaining the integrity of samples, particularly in a laboratory setting where precision is paramount [26]. Reagents like propanol, acetone, and xylene still impact costs. The greater usage of slide stickers may reflect the necessity for proper labeling and organization of specimens [27]. Optimal usage of tissue cassettes and slide stickers is crucial to avoid material wastage.

Overall, optimizing the use of these materials can enhance efficiency and ensure the accuracy of diagnostic processes. Continued monitoring of resource consumption will support effective laboratory management and improve operational outcomes. Strategies involving bulk purchases and careful inventory management could help reduce costs while maintaining adequate stock levels [28]. Strategic planning is key to maintaining a balance between resource utilization and cost management while ensuring high-quality diagnostic services [29,30].

The study performed an internal audit for one year in a single laboratory setting. Comprehensive studies are needed over a few years to provide a comparison of biopsy trends across quarters and resource utilization. This will help to decipher past trends and seasonal variations and plan for future demand. This manuscript tries to portray the reality and encourages the implementation of pathology lab administration to monitor material usage, optimize, and enhance the quality of reporting.

## Conclusions

Our study identifies several critical areas for improvement in the oral pathology laboratory's clinical and financial operations. The high volumes of excisional and incisional biopsies in our lab reflect an enhanced diagnostic workload, improving patient care and clinical throughput. However, this increased activity has also brought to light significant financial challenges, as noted in our financial deficit. The escalating cost of chemical reagents and the materials required for immunohistochemistry procedures are placing substantial strain on the laboratory budget. To ensure the department's long-term viability, implementation of cost management strategies, and improvement of operational efficiency with better resource allocation, strategic purchasing of reagents, and process optimization to reduce wastage must be ensured without compromising the quality of reporting.
